# Hypertension Surveillance in Rural Regions of Iran

**Published:** 2019-12

**Authors:** Mohammad Hossein PANAHI, Ali Reza MAHDAVI HEZAVEH, Tahereh SAMAVAT, Alieh HODJATZADEH, Elham YOUSEFI

**Affiliations:** 1. Ministry of Health and Medical Education, Tehran, Iran; 2. Department of Epidemiology and Biostatistics, School of Public Health, Tehran University of Medical Sciences, Tehran, Iran

## Dear Editor-in-Chief

Hypertension increases the risk of different cardiovascular diseases, including stroke, coronary artery disease, and heart failure. This disease accounts for 54% of stroke and 47% of ischemic kidney disease in the world (1–4). The prevalence of hypertension is expected to increase from 1 billion to 1.56 billion in 2025 (5). Hypertension accounts for 13% of deaths in the world (6). One-half of the patients were unaware of their illness (7). National program for prevention and control of hypertension with the aim of preventing and controlling hypertension and its complications in people over 30 yr of age was implemented in 1992 and was completely integrated into the health system in 2004. Two screening cycles were conducted in rural regions of Iran in 2004 and 2007 and they have remained opportunistic since 2011. In this program, the assessment of blood pressure was based on twice measurements with an interval of 1–2 min. Diagnosis was confirmed by frequent measurements and diagnosis of the doctor (8).

In this program, patients with hypertension are treated by a doctor once every three months. Based on the results of the surveillance program in rural regions in the first quarter of 2015 recorded in the portal of Department of Non-infectious Diseases of the Department of Health shows that from approximately 7 million rural residents recorded in the portal of this department, 635000 patients with hypertension are under treatment (Table 1). Seventy-one percent of these patients were examined by a doctor in the first quarter. Overall, 12,000 people with hypertension have a history of heart attack and stroke. About 11,000 people are also suffering from other complications.

**Table 1:** Results of hypertension surveillance rural population of Iran

***Sex***	***Population>30***	***Number of patients>30***	***Number of patients cared by GP***	***Number of CVD and stroke***	***Other complications***
Male	3425122	202437(5.91)^*^	138962 (68.64)	4996(2.47)	3967(1.96)
Female	3396140	431926(12.72)	309591 (71.68)	7029(1.63)	7454(1.73)
Total	6821262	634363(9.30)	448553 (70.71)	12025(1.90)	11421(1.80)

* Data are shown as frequency (percentage)

By lowering blood pressure cardiovascular risks such as myocardial infarction and stroke reduced by 20%–25% and 35%–40%, respectively (9). Appropriate health care programs namely, lifestyle modification and effective treatment can reduce hypertension and its complications (10).
